# Fatty acid supplementation during warming improves pregnancy outcomes after frozen blastocyst transfers: a propensity score-matched study

**DOI:** 10.1038/s41598-024-60136-0

**Published:** 2024-04-23

**Authors:** Ayano Sawado, Kenji Ezoe, Tetsuya Miki, Kazuki Ohata, Ayumi Amagai, Kiyoe Shimazaki, Tadashi Okimura, Keiichi Kato

**Affiliations:** Kato Ladies Clinic, 7-20-3 Nishishinjuku, Shinjyuku-ku, Tokyo, 160-0023 Japan

**Keywords:** Blastocyst transfer, Fatty acid, Outgrowth, Pregnancy outcomes, vitrification, Outcomes research, Translational research

## Abstract

This study aimed to examine the viability of human blastocysts after warming with fatty acids (FAs) using an in vitro outgrowth model and to assess pregnancy outcomes after a single vitrified-warmed blastocyst transfer (SVBT). For the experimental study, we used 446 discarded vitrified human blastocysts donated for research purposes by consenting couples. The blastocysts were warmed using FA‐supplemented (FA group) or non-FA-supplemented (control group) solutions. The outgrowth area was significantly larger in the FA group (*P* = 0.0428), despite comparable blastocyst adhesion rates between the groups. Furthermore, the incidence of outgrowth degeneration was significantly lower in the FA group than in the control group (*P* = 0.0158). For the clinical study, we retrospectively analyzed the treatment records of women who underwent SVBT in natural cycles between January and August 2022. Multiple covariates that affected the outcomes were used for propensity score matching as follows: 1342 patients in the FA group were matched to 2316 patients in the control group. Pregnancy outcomes were compared between the groups. The rates of implantation, clinical pregnancy, and ongoing pregnancy significantly increased in the FA group after SVBTs (*P* = 0.0091–0.0266). These results indicate that warming solutions supplemented with FAs improve blastocyst outgrowth and pregnancy outcomes after SVBTs.

## Introduction

With the development of vitrification techniques in assisted reproductive technologies, the use of a “freeze-all” strategy with freezing of the entire embryo cohort, followed by elective frozen embryo transfers (FETs) in subsequent cycles, has progressively increased^1–3^. In FET cycles, embryos can be transferred at the optimal time without the adverse impact of ovarian stimulation on uterine receptivity; therefore, pregnancy outcomes are improved after FETs compared to fresh embryo transfers^4–7^. In addition, FETs can reduce pregnancy and perinatal complications in the natural cycle^8^; thus, the demand for FETs is likely to continue to increase in the future. However, previous studies have demonstrated detrimental effects of vitrification on pre-implantation development and term pregnancy in oocytes and embryos^9–12^. Vitrification-induced impairment in developmental competence is caused by altered characteristics of the cytoplasmic organelles and cytoskeleton and increased abnormalities in chromosomal segregation^13–15^. Vitrification decreases intracellular lipid droplets (LDs), which participate in cellular membrane formation and play a versatile role as an energy source^16^, as well as competence during pre-implantation development^17^. Fatty acid (FA) supplementation in the warming solution restores LDs in the cytoplasm and improves developmental competence by promoting the β-oxidation pathway, which is crucial for oocyte maturation and early embryonic development^18–20^, in mice and bovines. Furthermore, in humans, FA supplementation during warming improved the developmental competence of cleavage-stage embryos, increasing outgrowth competence and pregnancy outcomes after frozen cleavage-stage embryo transfer^21^. However, it is unclear whether FA supplementation improves blastocyst viability after warming.

During pre-implantation development, dynamic transitions occur in metabolic reprogramming; the early-cleavage-stage embryos remain in a quiescent state that requires pyruvate and lactate and then transition into a highly oxidative state that needs glucose to support blastocyst development^22–24^. The LDs in early-cleavage-stage embryos are small and dispersed throughout the cytoplasm or may be partially clustered. However, larger LDs are formed after compaction, which provides an increased contact area with other organelles, such as mitochondria, facilitating lipid exchange, replenishment, and FA β-oxidation^18,25^. Furthermore, human embryos actively take up individual exogenous FAs at different rates at different stages of development^26^. The uptake of unsaturated FAs from the culture medium increases at the later stages of development, whereas saturated FAs are not actively taken up. A recent study reported that the lipid composition of blastocysts differs markedly from that at earlier developmental stages, since the biosynthesis of unsaturated fatty acids is activated at the blastocyst stage in mice and humans^27^. This activation is necessary for the formation of apical and basal domains to establish polarity toward blastocyst development. Therefore, LDs and FA metabolism are considered crucial at the blastocyst stage onward. In this study, we examined the effects of a commercial FA-supplemented warming solution (VT526; Kitazato Corporation, Supplemental Table 1) on blastocyst implantation competence using an in vitro outgrowth model. To evaluate the clinical efficacy of these solutions, we retrospectively analyzed pregnancy outcomes after single vitrified-warmed blastocyst transfers (SVBTs).

## Results

### In vitro experiments

#### LDs in blastocysts after warming

**Figure 1:**
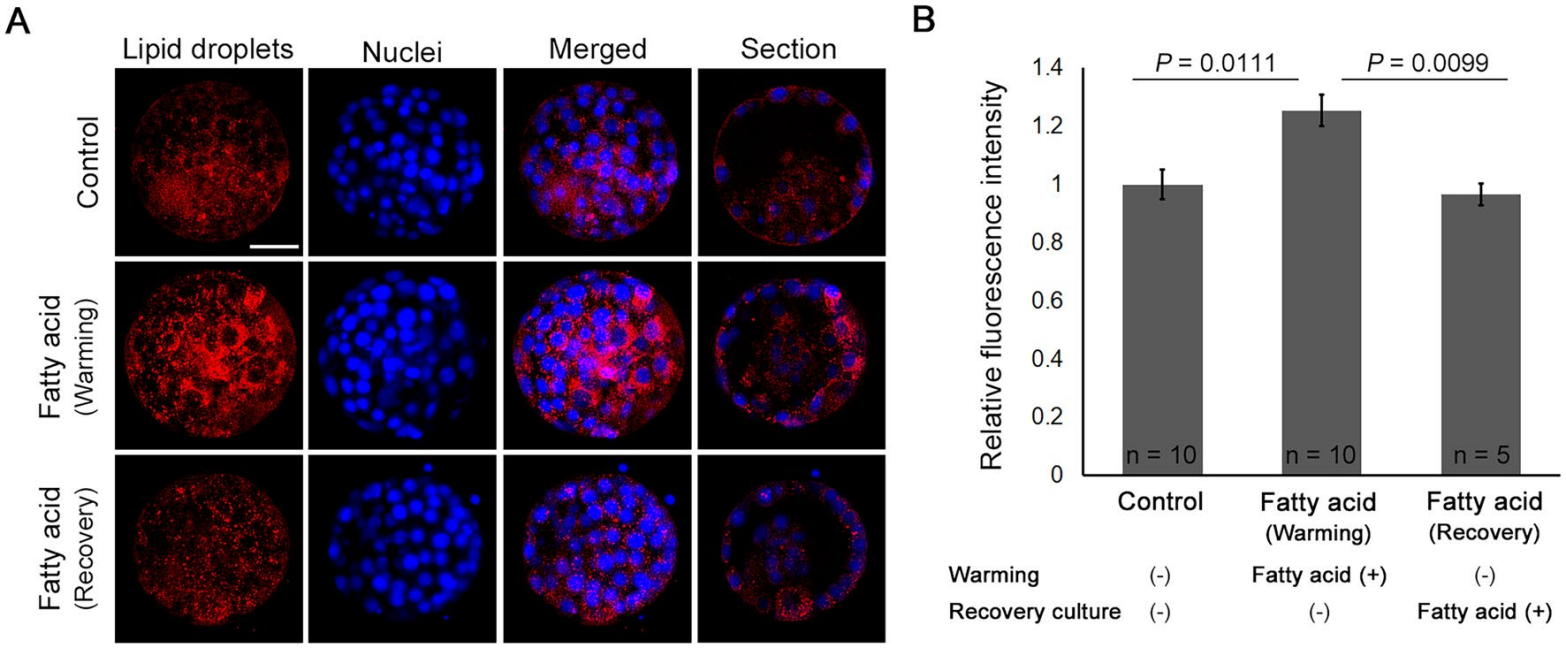
Lipid droplets in blastocysts after warming. (**A**) Representative images after Nile red staining; (**B**) data represent relative Nile red fluorescence intensities among blastocysts. In the control group, blastocysts were warmed without fatty acids (FAs) and cultured in a medium without FAs for 4 h (n = 10). In the FA (warming) group, blastocysts were warmed with FAs and cultured in a medium without FAs for 4 h (n = 10). In the FA (recovery) group, blastocysts were warmed without FAs and cultured in a medium with FAs for 4 h (n = 5). Scale bar represents 50 µm. Data are presented as mean ± standard error of the mean. Relative fluorescence was analyzed using ANOVA and Tukey’s test for post hoc analysis.

The distribution of LDs was examined using Nile red staining (Fig. 1A, Supplemental Table 2). The relative fluorescence intensity was significantly higher in blastocysts warmed with FAs than in those warmed without FAs (Fig. 1B). Furthermore, some blastocysts were warmed without FAs and then cultured in medium supplemented with FAs to examine whether FA supplementation during recovery culturing restores the LDs in blastocysts after warming. However, the relative fluorescence intensity of these blastocysts was comparable to that of blastocysts warmed and cultured without FAs.

#### Blastocyst outgrowth competence after warming with FA-supplemented solutions

**Figure 2:**
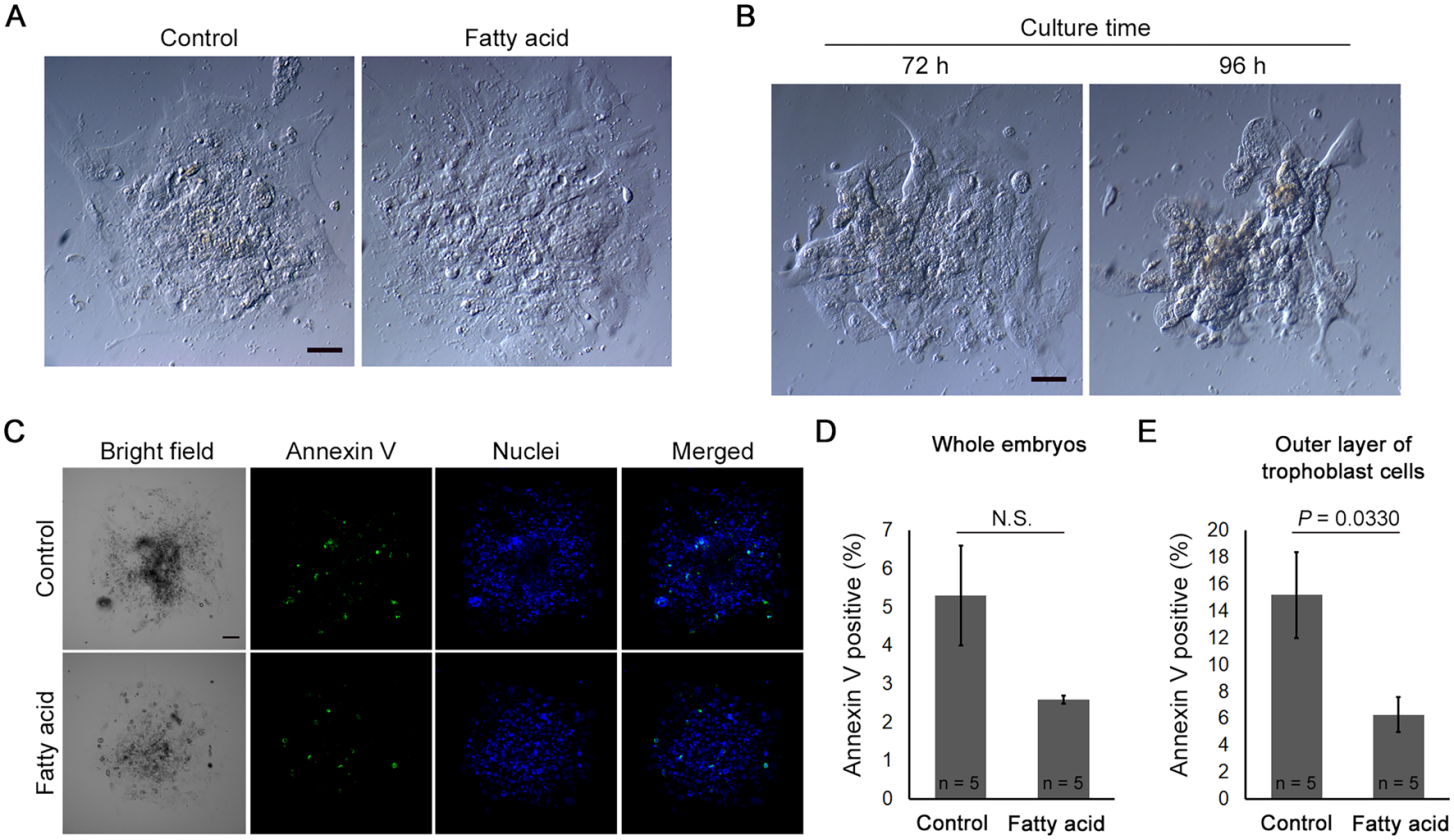
Trophoblast outgrowth and degeneration. (**A**) Blastocyst outgrowth after 96 h of culture; (**B)** outgrowth degeneration; (**C**) Annexin V staining for apoptotic cell detection (control group, n = 5, fatty acid group, n = 5); (**D** and **E**) the proportion of Annexin V-positive cells per whole embryo and per outer layer of trophoblast cells. Scale bar represents 50 µm. Data presented as mean ± standard error of the mean. The proportion of Annexin V-positive cells was compared using Student’s t-test.

Embryo characteristics, including maternal age, developmental speed, and blastocyst morphology, were comparable between the control and FA groups (Table 1). The adhesion rate after outgrowth culture in the FA group was comparable to that in the control group. However, the area of outgrowth was larger in the FA group than in the control group (Table 1 and Fig. 2A). Multivariate linear regression analysis demonstrated an increased outgrowth area after warming with FA supplementation (Table 2). Furthermore, the rate of outgrowth degeneration (Fig. 2B) was significantly lower in the FA group than in the control group. Although the proportion of Annexin V-positive cells in whole embryos was comparable between the control and FA groups, the proportion in the outer layer of trophoblast cells was significantly lower in the FA group than in the control group (Fig. 2C–E, Supplementary Table 2).

**Table 1:** Embryo characteristics and outgrowth outcomes.

	Control	Fatty acid	*P* value
No. of blastocysts, n	200	201	
Maternal age (y)	36.1 ± 0.3	36.2 ± 0.3	0.8088
< 35 y, n (%)	56 (27.6)	58 (27.9)	0.8044
35–37 y, n (%)	59 (29.1)	58 (27.9)	
38–40 y, n (%)	71 (35.0)	70 33.7)	
41–42 y, n (%)	14 (6.9)	15 (7.2)	
> 42 y, n (%)	0 (0)	0 (0)	
Culture time (h)	128.8 ± 0.8	128.2 ± 0.8	0.5962
Day 5, n (%)	98 (49.0)	105 (52.2)	0.5166
Day 6, n (%)	102 (51.0)	96 (47.8)	0.5166
Morphological grade of inner cell mass			0.3277
Grade A, n (%)	49 (24.5)	54 (26.9)	
Grade B, n (%)	70 (35.0)	80 (39.8)	
Grade C, n (%)	81 (40.5)	67 (33.3)	
Morphological grade of trophectoderm			0.9434
Grade A, n (%)	46 (23.0)	49 (24.4)	
Grade B, n (%)	71 (35.5)	71 (35.3)	
Grade C, n (%)	83 (41.5)	81 (40.3)	
Adhesion rate, n (%)			
24 h, n (%)	161 (80.5)	148 (73.6)	0.1019
48 h, n (%)	182 (91.0)	188 (93.5)	0.3424
72 h, n (%)	176 (88.0)	188 (93.5)	0.0556
96 h, n (%)	164 (82.0)	177 (88.1)	0.0890
Outgrowth area (10^4^ × µm^2^)			
24 h	1.8 ± 0.1	1.8 ± 0.1	0.9721
48 h	8.9 ± 0.5	8.8 ± 0.5	0.8688
72 h	18.5 ± 1.0	20.7 ± 1.0	0.1285
96 h	21.3 ± 1.3	25.2 ± 1.4	0.0428
Outgrowth degeneration, n (%)	62 (32.0)	41 (21.1)	0.0158

**Table 2:** Multivariate linear regression analysis for the outgrowth area.

	Coefficient of regression	95% confidential intervals		Standard error	T score	*P* value
		Lower	Upper			
Maternal age (y)*	− 273,851	− 442,770.0	− 104,931	85,919.4	− 3.19	0.0016
Culture time (h)*	− 8519.9	− 241,212.0	224,171.7	118,356.8	− 0.07	0.9427
Morphological grade of inner cell mass						
Grade A**	Reference	–	–	–	–	–
Grade B**	− 9794.3	− 35,282.0	15,693.2	12,964.1	− 0.76	0.4504
Grade C**	− 14,145.8	− 42,581.3	14,289.7	14,463.5	− 0.98	0.3287
Morphological grade of trophectoderm						
Grade A**	Reference	–	–	–	–	–
Grade B**	− 35,827.8	− 62,006.2	− 9649.4	13,315.4	− 2.69	0.0074
Grade C**	− 64,763.8	− 94,143.5	− 35,384.1	14,943.7	− 4.33	< 0.0001
Warming solutions						
Control**	Reference	–	–	–	–	–
Fatty acid**	19,826.4	1744.9	37,908.0	9197.0	2.16	0.0317

*Continuous variable (logit scale); **, categorical variable.

#### Sub-group analysis: maternal age

Blastocysts were categorized by maternal age according to the classification of the Society for Assisted Reproductive Technology^28^ (Table 1). Due to the low sample number, we combined the < 35 y and 35–37 y groups and the 38–40 y and 41–42 y groups for statistical analysis (Supplemental Table 3). The outgrowth area was comparable between the groups when maternal age was ≥ 38 years; nonetheless, it was significantly larger in the FA group when maternal age was < 38 years. Furthermore, the outgrowth degeneration rate decreased significantly after warming with FA-supplemented solutions when maternal age was < 38 years.

#### Sub-group analysis: developmental speed

Blastocysts were stratified by developmental speed because it is strongly correlated with pregnancy outcomes in the clinical setting^29^ (day 5 or 6; Supplemental Table 4). The adhesion rate of day 5 blastocysts was significantly higher in the FA group than in the control group. However, the adhesion rate of day 6 blastocysts was comparable between the groups. Furthermore, the outgrowth area of day 5 blastocysts was larger in the FA group, whereas the area of day 6 blastocysts was comparable between the groups. Outgrowth degeneration was not affected by FA supplementation in day 5 or 6 blastocysts.

#### Sub-group analysis: blastocyst morphology

Blastocysts were stratified according to morphology (good morphology: AA, AB, BA, and BB; poor morphology: AC, BC, CA, CB, and CC; Supplemental Table 5). The outgrowth area of blastocysts with good morphology was larger in the FA group than in the control group, despite the comparable outgrowth area of blastocysts with poor morphology between the groups. The outgrowth degeneration rate of blastocysts with good morphology was significantly lower in the FA group than in the control group.

### Mitochondrial membrane potential in warmed blastocysts

**Figure 3:**
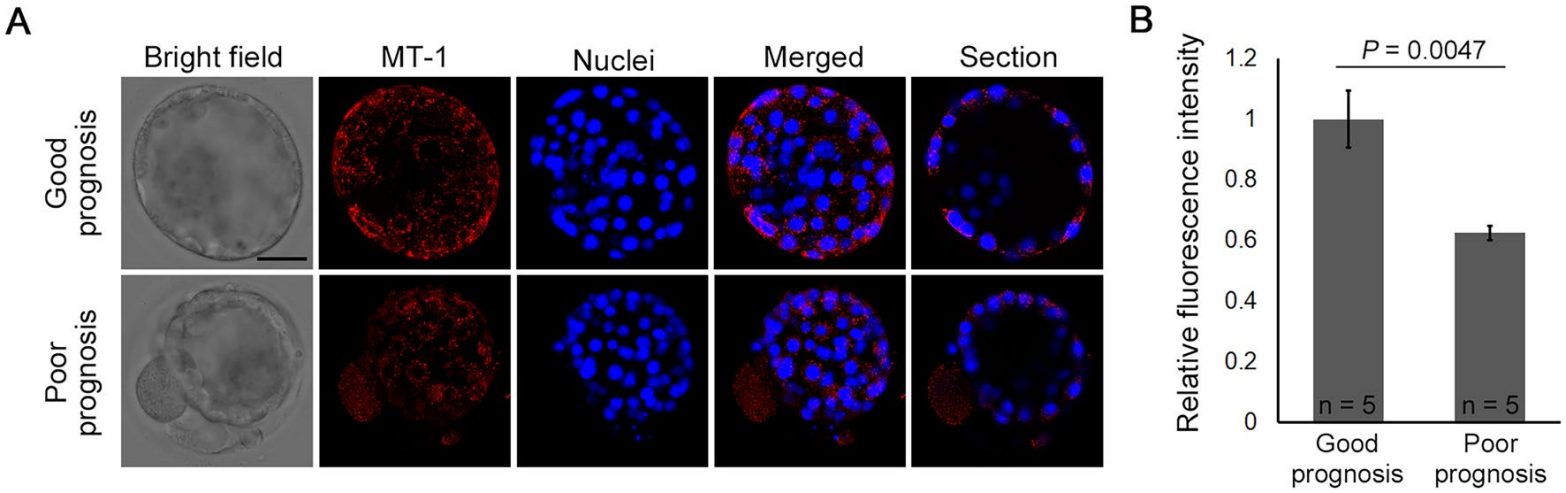
Mitochondrial membrane potential of blastocysts after warming. (**A**) Mitochondrial membrane potential of blastocysts with good prognosis and those with poor prognosis; (**B**) the fluorescence intensities of MT-1 dye (good prognosis, n = 5, poor prognosis, n = 5). Scale bar represents 50 µm. Data presented as mean ± standard error of the mean. Relative fluorescence was analyzed using Student’s t-test.

Mitochondrial membrane potential was compared between blastocysts with good prognosis and those with poor prognosis (Supplementary Table 2). A similar distribution of red fluorescence (MT-1) was observed in blastocysts with good versus poor prognosis (Fig. 3A). However, the relative fluorescence intensity was significantly higher in blastocysts with a good prognosis than in those with a poor prognosis (Fig. 3B).

### Retrospective cohort study

#### Patient characteristics

During the study period, the survival rates of blastocysts after warming were 99.5% (2316/2328) and 99.7% (1342/1346) in the control and FA groups, respectively (*P* = 0.3330). The number of patients and their characteristics, including the numbers of previous oocyte retrievals and embryo transfers and the endometrial thickness on the day of SVBT, differed between the groups (Table 3). After propensity score matching, 1324 patients were matched between the FA and control groups. Patient and embryo characteristics were comparable between the groups.

**Table 3:** Patient and cycle characteristics before and after propensity score matching.

	Before propensity score matching			After propensity score matching*		
	Control	Fatty acid	*P* value	Control	Fatty acid	*P* value
No. of patients, *n*	2316	1342		1324	1324	
Maternal age (years)	37.7 ± 0.1	37.6 ± 0.1	0.6420	37.6 ± 0.1	37.6 ± 0.1	0.5840
< 35 y, n (%)	530 (22.9)	331 (24.7)	0.5130	301 (22.7)	328 (24.8)	0.3736
35–37 y, n (%)	496 (21.4)	288 (21.5)		286 (21.6)	281 (21.2)	
38–40 y, n (%)	633 (27.3)	353 (26.3)		356 (26.9)	349 (26.4)	
41–42 y, n (%)	417 (18.0)	220 (16.4)		248 (18.7)	217 (16.4)	
> 42 y, n (%)	240 (10.4)	150 (11.2)		133 (10.1)	149 (11.3)	
Paternal age (years)	40.0 ± 0.1	39.6 ± 0.2	0.1038	39.8 ± 0.1	39.7 ± 0.2	0.5171
Previous OR cycles	2.7 ± 0.0	2.6 ± 0.0	0.0173	2.5 ± 0.0	2.6 ± 0.0	0.2797
Previous ET cycles	1.1 ± 0.0	0.9 ± 0.0	< 0.0001	0.9 ± 0.0	0.9 ± 0.0	1.0000
Previous implantation failure	0.8 ± 0.0	0.8 ± 0.0	0.1103	0.7 ± 0.0	0.7 ± 0.0	0.5007
Endometrial thickness on the day of SVBT (mm)	10.3 ± 0.0	10.2 ± 0.1	0.0235	10.2 ± 0.1	10.2 ± 0.1	0.9455
Culture time to the expanded blastocyst stage (h)	125.8 ± 0.3	125.6 ± 0.3	0.5964	125.7 ± 0.3	125.6 ± 0.3	0.7503
Morphological grade						
ICM Grade A, *n* (%)	937 (40.5)	575 (42.9)	0.2563	572 (43.2)	563 (42.5)	0.9232
Grade B, *n* (%)	819 (35.4)	470 (35.0)		464 (35.1)	466 (35.2)	
Grade C, *n* (%)	560 (24.2)	297 (22.1)		288 (21.8)	295 (22.3)	
TE Grade A, *n* (%)	768 (33.2)	446 (33.2)	0.9945	436 (32.9)	444 (33.5)	0.6620
Grade B, *n* (%)	644 (27.8)	371 (27.7)		387 (29.2)	366 (27.6)	
Grade C, *n* (%)	904 (33.2)	525 (39.1)		501 (37.8)	514 (38.8)	

The data are shown as the mean and standard error of the mean, unless otherwise indicated.

*Covariates: previous OR cycles, previous embryo transfers, and endometrial thickness on the day of SVBT.

OR, oocyte retrieval; ET, embryo transfer; SVBT, single vitrified-warmed blastocyst transfer; ICM, inner cell mass; TE, trophectoderm.

#### Pregnancy outcomes after SVBTs

Implantation, clinical pregnancy, and ongoing pregnancy rates were significantly higher in the FA group than in the control group (Table 4). Multivariate logistic regression analysis demonstrated that warming with FA-supplemented solutions significantly improved pregnancy rates (Supplemental Table 6). However, the rates of early pregnancy loss and miscarriage in the first trimester were comparable between the groups.

**Table 4:** Pregnancy outcomes after single vitrified-warmed blastocyst transfers.

	Control	Fatty acid	*P* value
Patients, n	1324	1324	
Embryo transfer cycles, *n*	1324	1324	
Implantation, *n* (%)	611 (46.2)	668 (50.5)	0.0266
Clinical pregnancies, *n* (%)	552 (41.7)	619 (46.8)	0.0088
Ongoing pregnancies, *n* (%)	465 (35.1)	530 (40.0)	0.0091
Early pregnancy loss, *n* (%)	59 (9.7)	49 (7.4)	0.1447
Miscarriages in the first trimester, *n* (%)	87 (15.8)	90 (14.6)	0.5841

#### Sub-group analysis: maternal age

The patients were categorized by maternal age according to the Society for Assisted Reproductive Technology classification^28^ (Table 3). Owing to the low sample number, we combined the 41–42 y and > 42 y groups for statistical analysis (Supplemental Table 7). Although the ongoing pregnancy rates were higher when the patients were 40 years or younger than when they were 40 years old, the difference was not significant.

#### Sub-group analysis: developmental speed

Patients were stratified according to developmental speed (day 4 or 5, OR 6 or 7; Supplemental Table 8). The ongoing pregnancy rate of day 4 or 5 blastocysts was significantly higher in the FA group than in the control group. In contrast, the pregnancy rates of day 6 or 7 blastocysts were comparable between the groups.

#### Sub-group analysis: blastocyst morphology

Patients were stratified according to blastocyst morphology (good morphology: AA, AB, BA, and BB; poor morphology: AC, BC, CA, CB, and CC; Supplemental Table 9). The rates of clinical and ongoing pregnancies for blastocysts with good morphology were significantly higher in the FA group than in the control group. In contrast, the rates of clinical and ongoing pregnancies for blastocysts with poor morphology were comparable between the groups.

## Discussion

FA-supplemented warming solutions increase the LDs of blastocysts and promote outgrowth in vitro by decreasing cell apoptosis during outgrowth culturing. Furthermore, in the clinical setting, the rates of implantation, clinical pregnancy, and ongoing pregnancy improve significantly after the transfer of blastocysts warmed with FA-supplemented solutions.

We first demonstrated that warming of blastocysts with the FA-supplemented solution recovered the LDs in the cytoplasm, whereas LDs were not increased in blastocysts when the recovery culture did not contain FAs. A previous study reported that vitrification caused transient membrane damage and allowed an impermeable solute, 4 kDa fluorescein isothiocyanate–dextran, to enter the cytoplasm^30^. Therefore, this may be a reason why adding FAs into the warming solution more effectively compensates with LDs than FA supplementation during recovery culturing.

We examined the effects of FA supplementation on blastocyst adhesion. To avoid the influence of maternal factors, such as the endometrial environment, we used an in vitro outgrowth model, a method used to evaluate blastocyst adhesion competence^31^. The FA-supplemented solution promoted blastocyst outgrowth. Furthermore, warming with the solution reduced the incidence of apoptosis, especially in the outer layer of trophoblast cells, following outgrowth degeneration. However, the molecular mechanisms by which FA-supplemented warming solutions promote outgrowth and reduce apoptosis remain unclear. FA supplementation may restore intracytoplasmic LDs^17^ and prevent the shortage of energy sources at a later stage of outgrowth culturing, reducing apoptosis in trophoblast cells.

FA-supplemented warming solutions were effective in young patients, day 5 blastocysts, and morphologically good blastocysts, but not in older patients, day 6 blastocysts, or morphologically poor blastocysts. Mitochondrial function controls mitochondrial FA β-oxidation, which is the primary metabolic pathway for FA degradation and the maintenance of energy homeostasis, and subsequent ATP production^32^. Mitochondrial function is reportedly associated with maternal age, embryo development, and embryo quality^33,34^; in fact, the present study demonstrated that blastocysts with poor prognosis exhibited lower mitochondrial membrane potential than blastocysts with good prognosis. As such, β-oxidation may not be activated in blastocysts produced in older patients, day 6 blastocysts, and morphologically poor blastocysts, despite sufficient metabolic substances, such as FA, in the embryo. Therefore, poor blastocyst outgrowth after warming with FA-supplemented solutions can be attributed to low mitochondrial function. Improving mitochondrial function in blastocysts with poor prognosis may be one way to maximize the benefit of FA-supplemented solutions. Further molecular studies are required to determine the differences in the effects of FA-supplemented solutions on blastocyst outgrowth between blastocysts with good and poor prognoses.

We conducted a retrospective cohort study using propensity score matching. The rates of implantation, clinical pregnancy, and ongoing pregnancy after SVBTs increased significantly upon using a FA-supplemented solution. Furthermore, the FA-supplemented solution was more effective in cases with good prognosis (day 4 or 5 blastocysts and morphologically good blastocysts) than in cases with poor prognosis (day 6 or 7 blastocysts and morphologically poor blastocysts), similar to the outcomes of the in vitro outgrowth model. Notably, the outgrowth degeneration rate decreased in blastocysts produced in young patients and in morphologically good blastocysts upon using the FA-supplemented solution. Furthermore, the rate of early pregnancy loss decreased in these blastocysts after warming with the FA-supplemented solution, suggesting that outgrowth degeneration may reflect the possibility of early pregnancy loss.

It has been established that the exogenous supply of FAs in culture medium can affect reproductive potential, such as oocyte maturation, cryopreservation, oxidative stress, and cellular signaling, in various species^35–37^. Furthermore, an excessive amount of FAs, especially saturated FAs, reportedly resulted in reticulum stress, lower fertilization and embryo development rates, and caused irreversible damage to fetal growth and offspring health^38–43^. However, the concentration of FAs in the warming solution used in this study was much lower (approximately 1/1000) than those in previous studies that demonstrated the detrimental effects of FAs. Furthermore, prolonged cellular exposure to high levels of extracellular lipids is reported to result in excess peroxidation of free FA, leading to the production of excess reactive oxygen species and toxic lipid peroxides, which together are known as lipotoxicity^44,45^. However, our previous study reported that the amount of LDs remained significantly lower in vitrified oocytes than in fresh oocytes although an FA-supplemented solution was used, suggesting that FA-supplemented warming solutions are not associated with lipotoxicity and decreased blastocyst viability. The present study demonstrated an improvement in blastocyst outgrowth and pregnancy outcomes after SVBTs. Further studies on maternal and obstetric outcomes after SVBT are needed to validate the safety of these warming solutions.

The strength of this study is that we performed both experimental and clinical research and demonstrated concordant outcomes. Furthermore, we examined a large clinical dataset obtained from a single center, and the culture conditions and SVBT procedures were uniform. Propensity score matching was performed to balance the baseline characteristics of the control and FA groups. Therefore, it was not necessary to consider the bias caused by differences in the potential conditions. Moreover, we included patient age, embryonic development speed, and blastocyst morphological grade as confounders significantly associated with ongoing pregnancy rate after SVBTs in the multivariate logistic regression analysis. In addition, we conducted a power analysis between the control and FA groups and found a power of 99.9% for the incidence of ongoing pregnancy.

However, this study had certain limitations. In the experimental study, we evaluated the viability of trophoblast cells using an outgrowth model; however, the viability of epiblast cells was not assessed. Further molecular studies are required to evaluate the health of peri-implantation embryos. In addition, this clinical study was retrospective, necessitating further multicenter studies to ascertain the generalizability of the findings to other clinics with different protocols and/or patient demographics. Despite using propensity score matching and multivariate models to adjust for confounders, not all possible confounders, including uterine environment and patient conditions, could be adjusted for. Furthermore, maternal and perinatal outcomes after warming with FA‐supplemented solutions should be assessed. The lipids that are decreased by vitrification procedures should also be determined, since strategies to supplement the deficiency of these specific lipids are essential to minimize cell damage and maintain cell stability caused by their reduction.

In conclusion, FA‐supplemented warming solutions improved pregnancy outcomes after SVBTs, particularly in patients with a good prognosis. These warming solutions could contribute to maximizing clinical outcomes, shortening treatment time, and reducing patient burden. However, continuous development of vitrification techniques is required to improve the clinical outcomes of older patients in whom there could be further compromise of cellular structure and metabolism.

## Methods

### approval

This experimental and retrospective cohort study was approved by the Institutional Review Board of Kato Ladies Clinic (approval numbers: 21–24, 23–02). All procedures were performed in accordance with the ethical standards of the responsible committee on human experimentation (institutional and national) and the Helsinki Declaration of 1964 and its later amendments. Written informed consent for the retrospective use of data was obtained from all patients.

### Blastocyst warming

We used 401 discarded vitrified human blastocysts generously donated for research purposes by consenting couples who had conceived babies after completing fertility treatment (Table 1). The embryos were vitrified at the blastocyst stage on day 5 or 6. The donated blastocysts were randomly allocated to two groups depending on the type of warming solution used: FA‐supplemented solution (VT526; Kitazato Corporation, Supplemental Table 1) and non-supplemented solution (VT506; Kitazato Corporation, Shizuoka, Japan), denoted as the FA and control groups, respectively. Warming procedures were performed using the Cryotop method^17,21^. Briefly, the tip of the Cryotop was dipped in a warming solution (thawing solution) at 37 °C for 1 min, and the warmed embryos were transferred to a diluent solution. After 3 min, the samples were transferred to a washing solution for 5 min, and the embryo was subsequently transferred to washing solution 2 for an additional 1 min.

## LD staining

Vitrified blastocysts were warmed with (n = 10) or without (n = 15) FAs and cultured in ONESTEP medium (Nakamedical, Tokyo, Japan) for 4 h (Supplemental Table 2). Five blastocysts warmed without FA were cultured in ONESTEP medium supplemented with 1% Chemically Defined Lipid Concentrate (Thermo Fisher Scientific, MA, USA). The LDs in blastocysts were examined by Nile red staining, as previously reported^17^. Briefly, blastocysts were fixed in a 10% formalin neutral buffer solution (Fujifilm Wako Pure Chemical, Tokyo, Japan) for 1 h at 26–28 °C. The blastocysts were transferred to phosphate-buffered saline (PBS) containing 0.2 µL/mL Nile Red (Fujifilm Wako Pure Chemical) and cultured for 30 min at 26–28 °C. The blastocysts were transferred to PBS containing 1 mg/mL Hoechst 33,342 (Sigma-Aldrich, St. Louis, MO, USA) and incubated for 30 min to stain the nuclei. The blastocysts were imaged using a BZ-X800 fluorescence microscope (Keyence, Osaka, Japan). The fluorescence intensity was measured using the BZ-X800 Analyzer software (Keyence), as previously reported^46^.

### Blastocyst outgrowth

We investigated the proportions of adhered blastocysts and outgrowth areas to estimate the implantation competence of blastocysts in vitro. We performed an outgrowth assay as previously described^46,47^. Briefly, we pre-coated culture dishes with 10 μg/mL of fibronectin (Sigma-Aldrich) overnight at 4 °C. Subsequently, we pipetted a 20 μL drop of ONESTEP medium (Nakamedical) and added it onto the slide before adding the oil overlay. After removing the zona pellucida using acid Tyrode's solution, we placed the blastocysts individually in drops and cultured them for 96 h in a humidified incubator at 37 °C with 5% O_2_, 5% CO_2_, and 90% N_2_. Trophoblasts grew outward from the blastocysts and became visible. Subsequently, we designated these embryos as adhesion-initiating blastocysts. We measured the outgrowth area at the end of culturing using NIS-Elements imaging software (version 2.0; Nikon, Tokyo, Japan). Briefly, we selected the outer edge of the trophoblast and automatically calculated the outgrowth area. We designated the embryos as degenerated when the outgrowth area decreased by more than 10% compared to the maximum area of outgrowth^47^. We further stratified the outgrowth outcomes by maternal age, developmental speed, and blastocyst morphology and evaluated them using Gardner’s criteria^48^ for sub-group analysis.

### Apoptosis detection

Apoptotic cells in outgrowth embryos were detected using the Annexin V-FITC Apoptosis Detection Kit (Sigma-Aldrich) according to the manufacturer’s instructions. Briefly, the embryos were incubated with Annexin V-FITC Conjugate diluted by 1 × Binding Buffer (1:100) for 10 min at 26–28 °C, washed with PBS, and incubated in PBS containing 1 mg/mL Hoechst 33,342 for 30 min at 26–28 °C. The embryos were imaged using a BZ-X800 fluorescence microscope, and the numbers of total cells, outer layers of trophoblast cells, and Annexin V-positive cells were counted.

### Assay of mitochondrial membrane potential

Mitochondrial membrane potential was monitored using the MT-1 MitoMP Detection Kit (Dojindo Laboratories, Kumamoto, Japan) according to the manufacturer’s instructions. Briefly, blastocysts were incubated in ONESTEP medium supplemented with 0.1% MT-1 Dye for 30 min at 37 °C and washed with ONESTEP medium. The blastocysts were then incubated in PBS supplemented with Hoechst 33,342 to stain the nuclei. Images were acquired using a BZ-X800 microscope.

### Retrospective cohort study

#### Patients

We reviewed the records of 4426 treatment cycles from 4426 women who underwent SVBTs in natural cycles between January 2023 and August 2023. We excluded 118 patients who underwent preimplantation genetic testing and 650 patients with recurrent implantation failure (four or more unsuccessful ETs^49^). This study included only one cycle per patient.

#### Study design

From January 2023 to May 2023, we warmed the vitrified blastocysts using non‐supplemented solutions, representing the control group (VT506; Kitazato Corporation). From June 2023 to August 2023, we warmed them using FA‐supplemented solutions (VT526; Kitazato Corporation), representing the FA group. We performed propensity score matching using JMP software (SAS, Cary, NC, USA) to reduce bias in patient characteristics^50,51^. Briefly, we used multiple covariates, including previous oocyte retrieval cycles, embryo transfers, and endometrial thickness on the day of SVBT, for propensity score matching. We matched each patient who obtained surviving blastocysts after warming in the FA group with controls in a 1:1 ratio. Using caliper matching, each patient who had similar propensity scores within a caliper of 0.05 was matched. We compared pregnancy outcomes after SVBTs between the control and FA groups.

#### Minimal ovarian stimulation, oocyte retrieval, and embryo culture

The protocols for ovarian stimulation, oocyte retrieval, and embryo culture were uniform throughout the study period. Clomiphene citrate (CC)-based minimal stimulation was performed as previously reported^4,52^. Briefly, CC was orally administered with an extended regimen (50 mg/day), starting on day 3 of the OR cycle and continuing until the day before the induction of final oocyte maturation. Ovulation was triggered by a nasal spray containing the gonadotropin-releasing hormone agonist, buserelin (450 μg). A minimal dose of human menopausal gonadotropin or recombinant follicle-stimulating hormone was administered on days 8 and 10 to induce final follicular growth and maturation.

Oocyte retrieval was performed 34–36 h after triggering using a 21-G needle (Kitazato Corporation). Cumulus-oocyte complexes (COCs) were collected, washed, and transferred to human tubal fluid (HTF) medium (Kitazato Corporation) under paraffin oil at 37 °C (gas phase: 5% O_2_, 5% CO_2_, and 90% N_2_) until either conventional IVF 3 h later or denudation in cases of intracytoplasmic sperm injection (ICSI) 4 h after oocyte retrieval. The oocytes were denuded and cultured in HTF medium prior to ICSI^53^.

Sperm samples were collected by masturbation and washed by centrifugation through 70% and 90% density gradients (Isolate; Irvine Scientific, Santa Ana, CA, USA). The prepared sperm samples were cultured in HTF medium at 37 °C (gas phase: 5% O_2_, 5% CO_2_, and 90% N_2_) until use.

For conventional IVF, HTF medium supplemented with 10% serum substitute (Irvine Scientific) was used as the fertilisation medium^54^. COCs were cultured with sperm (100,000 sperm/mL) under 5% CO_2_ at 37 °C. Extrusion of the second polar body was confirmed 5 h after insemination (day 0), following the removal of cumulus cells. The oocytes were individually cultured in EmbryoSlide (Vitrolife, Inc., Göteborg, Sweden), which is suitable for group culture, in 180 µL of medium drop (ONESTEP medium; Nakamedical, Inc., Tokyo, Japan) under paraffin oil. In cases of ICSI, oocytes were inseminated in HEPES-buffered HTF medium^55,56^. The injected oocytes were immediately placed onto EmbryoSlides and cultured individually in 180 µL of medium under paraffin oil. Embryos were cultured at 37 °C (gas phase: 5% O_2_, 5% CO_2_, and 90% N_2_) in an Embryoscope + time-lapse incubator (Vitrolife) for 2 days. Fertilisation was observed using EmbryoViewer software (Vitrolife).

The morphological grades of the inner cell mass and TE in blastocysts were evaluated according to Gardner’s criteria^48^.

#### Blastocyst cryopreservation

Cryotop (Kitazato Corporation) was used for embryo vitrification, as described previously^17,21^. Briefly, blastocysts were equilibrated in an equilibration solution composed of 7.5% (volume/volume) ethylene glycol and 7.5% (volume/volume) dimethylsulfoxide for 15 min. The blastocysts were then transferred to a vitrification solution containing 15% (volume/volume) ethylene glycol, 15% (volume/volume) dimethylsulphoxide, and 0.5 M sucrose for 1.5 min. Next, the blastocysts were placed on the Cryotop and immediately plunged into liquid nitrogen. Warming was performed as described above.

#### SVBT procedure

In the present study, the administration of a gonadotropin-releasing hormone agonist to induce final oocyte maturation and ovulation was the only pharmacological intervention^57^. Monitoring included transvaginal ultrasonography and blood hormone tests. When the leading follicle attained a diameter of 18 mm, ovulation was triggered via buserelin treatment. SVBTs were performed under vaginal ultrasound guidance using a specially designed soft silicone inner catheter (Kitazato ET catheter; Kitazato Corporation). A single embryo was placed in a minimal volume in the upper part of the uterine cavity^51^. Dydrogesterone (Duphaston, 30 mg/day; Mylan EPD G.K., Tokyo, Japan) was administered orally during the early luteal phase after SVBT.

Implantation was defined by serum human chorionic gonadotropin levels (≥ 20 mIU/mL)^58^. Clinical and ongoing pregnancy rates were defined by ultrasonographic observation of the gestational sac 3 weeks after SVBTs and the fetal heartbeat 5 weeks after SVBTs^59^. Early pregnancy loss and miscarriage in the first trimester were defined as the absence of a gestational sac after implantation and live birth after confirmation of a gestational sac^51^.

### Statistical analysis

We performed statistical analyses using the JMP software. The proportions of the data were analyzed using the chi-square test and Fisher’s exact test. Continuous parameters were compared using Student’s t-test or ANOVA, and significance was determined using Tukey’s test for post hoc analysis when normality could be accepted; otherwise, the Mann–Whitney U test was used. Univariate regression analysis was used to identify confounders potentially associated with the outcomes. The likelihood ratio test for the significance of the coefficient was performed, and the variables with *P* < 0.05 were used as confounders (Supplemental Tables 10 and 11). We performed multiple linear regression analysis to assess the relative importance of possible predictor variables in explaining the area of blastocyst outgrowth. Moreover, we performed multivariate logistic regression analysis to adjust for bias and verify statistical significance. Adjusted odds ratios are reported with 95% confidence intervals for each group. The goodness of fit of the multivariate logistic regression analysis was evaluated using the Pearson Chi-square statistic. We set statistical significance at *P* < 0.05.

### Supplementary Information

Supplementary Tables.

## Data Availability

The datasets generated and/or analyzed during the current study are not publicly available for the protection of the participants but are available from the corresponding author upon reasonable request.
