# Supplementary material for: Fatty acid supplementation during warming improves pregnancy outcomes after frozen blastocyst transfers: a propensity score-matched study
Source: Sci Rep. 2024 Apr 23;14:9343. doi: 10.1038/s41598-024-60136-0 (PMC11039611; doi:10.1038/s41598-024-60136-0)
Supplement: Supplementary file 1 — Supplementary Tables. [file 41598_2024_60136_MOESM1_ESM.docx]

**Fatty acid supplementation during warming improves pregnancy outcomes after frozen blastocyst transfers: a propensity score-matched study**

Ayano Sawado^1§^, Kenji Ezoe^1§*^, Tetsuya Miki^1^, Kazuki Ohata^1^, Ayumi Amagai^1^, Kiyoe Shimazaki^1^, Tadashi Okimura^1^, Keiichi Kato^*1^

^1^Kato Ladies’ Clinic, Tokyo, Japan

^§^ A. Sawado and K. Ezoe contributed equally to this manuscript.

**^*^Corresponding author:**

Keiichi Kato, MD, PhD

Kato Ladies Clinic, 7-20-3 Nishishinjuku, Shinjyuku-ku, Tokyo, 160-0023, Japan

Tel: +81-3-3366-3777

Fax: +81-3-3366-3908

E-mail: k-kato@towako.net

Kenji Ezoe, PhD

Kato Ladies Clinic, 7-20-3 Nishishinjuku, Shinjyuku-ku, Tokyo, 160-0023, Japan

Tel: +81-3-3366-1073

Fax: +81-3-3366-3908

E-mail: k-ezoe@towako-kato.com

**Supplemental Table 1.** **Fatty acid composition in warming solutions**

| Components of fatty acid |  | Length of fatty acids | Molecular weight (g/mol) | Concentration (nM) |
| --- | --- | --- | --- | --- |
| Unsaturated fatty acid | Arachidonic acid | Long-chain | 304.47 | 65.7 |
|  | Linoleic acid | Long-chain | 280.4472 | 356.6 |
|  | Linolenic acid | Long-chain | 278.43 | 359.2 |
|  | Oleic acid | Long-chain | 282.46 | 354.0 |
|  | Palmitoleic acid | Long-chain | 254.414 | 393.0 |
| Saturated fatty acid | Myristic acid | Long-chain | 228.37 | 437.9 |
|  | Palmitic acid | Long-chain | 256.43 | 390.0 |
|  | Stearic acid | Long-chain | 284.48 | 351.5 |

**Supplemental Table 2. Characteristics of embryos used for Nile red staining, Annexin V staining, and mitochondrial membrane potential monitoring**

|  | Staining for lipid droplet | | | |  | Apoptosis detection | | |  | Monitoring for mitochondrial membrane potential | | |  |
| --- | --- | --- | --- | --- | --- | --- | --- | --- | --- | --- | --- | --- | --- |
|  | Control | Fatty acid  (Warming) | Fatty acid  (Recovery) | *P* value |  | Control | Fatty acid | *P* value |  | Good prognosis | Poor prognosis | *P* value | |
| No. of blastocysts, n | 10 | 10 | 5 |  |  | 5 | 5 |  |  | 5 | 5 |  | |
| Maternal age (y) | 34.0 ± 0.6 | 34.2 ± 1.0 | 33.5 ± 0.8 | 0.8173 |  | 35.2 ± 0.2 | 35.4 ± 0.2 | 0.5447 |  | 32.2 ± 0.6 | 41.6 ± 0.7 | < 0.0001 | |
| Culture time (h) | 116.5 ± 0.8 | 119.0 ± 1.9 | 118.9 ± 0.8 | 0.1424 |  | 116.0 ± 0.7 | 117.6 ± 1.1 | 0.2623 |  | 118.0 ± 0.8 | 140.8 ± 1.6 | < 0.0001 | |
| Day 5, n (%) | 10 (100) | 10 (100) | 5 (100) | – |  | 5 (100) | 5 (100) | – |  | 5 (100) | 0 (0) | 0.0016 | |
| Day 6, n (%) | 0 (0) | 0 (0) | 0 (0) | – |  | 0 (0) | 0 (0) | – |  | 0 (0) | 5 (100) | 0.0016 | |
| Morphological grade of ICM |  |  |  | 0.8779 |  |  |  | 0.2918 |  |  |  | 0.0016 | |
| Grade A, n (%) | 7 (70.0) | 6 (60.0) | 3 (60.0) |  |  | 4 (80.0) | 5 (100) |  |  | 5 (100) | 0 (0) |  | |
| Grade B, n (%) | 3 (30.0) | 4 (40.0) | 2 (40.0) |  |  | 1 (20.0) | 0 (0) |  |  | 0 (0) | 0 (0) |  | |
| Grade C, n (%) | 0 (0) | 0 (0) | 0 (0) |  |  | 0 (0) | 0 (0) |  |  | 0 (0) | 5 (100) |  | |
| Morphological grade of TE |  |  |  | 0.7554 |  |  |  | 1.0000 |  |  |  | 0.0016 | |
| Grade A, n (%) | 5 (50.0) | 4 (40.0) | 3 (60.0) |  |  | 2 (40.0) | 2 (40.0) |  |  | 5 (100) | 0 (0) |  | |
| Grade B, n (%) | 5 (50.0) | 6 (60.0) | 2 (40.0) |  |  | 3 (60.0) | 3 (60.0) |  |  | 0 (0) | 0 (0) |  | |
| Grade C, n (%) | 0 (0) | 0 (0) | 0 (0) |  |  | 0 (0) | 0 (0) |  |  | 0 (0) | 5 (100) |  | |

ICM, inner cell mass; TE, trophectoderm.

**Supplemental Table 3. Embryo characteristics and outgrowth outcomes, stratified by maternal age**

| Female age | < 38 years | | |  | ≥ 38 years | | |
| --- | --- | --- | --- | --- | --- | --- | --- |
|  | Control | Fatty acid | *P* value |  | Control | Fatty acid | *P* value |
| No. of blastocysts, n | 115 | 116 |  |  | 85 | 85 |  |
| Maternal age (y) | 33.7 ± 0.3 | 33.9 ± 0.3 | 0.6087 |  | 39.4 ± 0.1 | 39.4 ± 0.1 | 0.8493 |
| Culture time (h) | 128.8 ± 1.1 | 128.5 ± 1.0 | 0.7983 |  | 128.7 ± 1.2 | 127.8 ± 1.3 | 0.6107 |
| Day 5, n (%) | 56 (48.7) | 59 (51.3) | 0.9464 |  | 42 (49.4) | 48 (56.5) | 0.3566 |
| Day 6, n (%) | 57 (49.1) | 59 (50.9) |  |  | 43 (50.6) | 37 (43.5) |  |
| Morphological grade of inner cell mass |  |  | 0.2430 |  |  |  | 0.8925 |
| Grade A, n (%) | 26 (22.6) | 32 (27.6) |  |  | 23 (27.1) | 22 (25.9) |  |
| Grade B, n (%) | 39 (33.9) | 46 (39.7) |  |  | 31 (36.5) | 34 (40.0) |  |
| Grade C, n (%) | 50 (43.5) | 38 (32.8) |  |  | 31 (36.5) | 29 (34.1) |  |
| Morphological grade of trophectoderm |  |  | 0.7315 |  |  |  |  |
| Grade A, n (%) | 22 (19.1) | 56 (22.4) |  |  | 24 (28.2) | 23 (27.1) |  |
| Grade B, n (%) | 40 (34.8) | 42 (36.2) |  |  | 31 (36.5) | 29 (34.1) |  |
| Grade C, n (%) | 53 (46.1) | 48 (41.4) |  |  | 30 (35.3) | 33 (38.8) |  |
| Adhesion at 96 h, n (%) | 95 (82.6) | 103 (88.8) | 0.1793 |  | 69 (81.2) | 74 (87.1) | 0.2941 |
| Outgrowth area at 96 h (10⁴×㎛²) | 22.1 ± 0.2 | 27.1 ± 0.2 | 0.0468 |  | 20.3 ± 0.2 | 23.2 ± 0.2 | 0.3387 |
| Outgrowth degeneration, n (%) | 34 (30.4) | 20 (17.9) | 0.0287 |  | 28 (34.2) | 21 (25.6) | 0.2324 |

**Supplemental Table 4. Embryo characteristics and outgrowth outcomes, stratified by developmental speed**

| Day of blastocyst | Day 5 | | |  | Day 6 | | |
| --- | --- | --- | --- | --- | --- | --- | --- |
|  | Control | Fatty acid | *P* value |  | Control | Fatty acid | *P* value |
| No. of blastocysts, n | 98 | 105 |  |  | 102 | 96 |  |
| Maternal age (y) | 36.2 ± 0.4 | 36.5 ± 0.4 | 0.6535 |  | 36.1 ± 0.4 | 36.0 ± 0.3 | 0.8575 |
| Culture time (h) | 117.7 ± 0.3 | 118.3 ± 0.4 | 0.2500 |  | 139.0 ± 0.3 | 139.0 ± 0.5 | 0.4597 |
| Morphological grade of inner cell mass |  |  | 0.9771 |  |  |  | 0.1378 |
| Grade A, n (%) | 37 (37.8) | 39 (37.1) |  |  | 12 (11.8) | 15 (15.6) |  |
| Grade B, n (%) | 38 (38.8) | 40 (38.1) |  |  | 32 (31.4) | 40 (41.7) |  |
| Grade C, n (%) | 23 (23.5) | 26 (24.8) |  |  | 58 (56.9) | 41 (42.7) |  |
| Morphological grade of trophectoderm |  |  | 0.3279 |  |  |  | 0.4765 |
| Grade A, n (%) | 35 (35.7) | 36 (34.3) |  |  | 11 (10.8) | 13 (13.5) |  |
| Grade B, n (%) | 46 (47.0) | 42 (40.0) |  |  | 25 (24.5) | 29 (30.2) |  |
| Grade C, n (%) | 17 (17.4) | 27 (25.7) |  |  | 66 (64.7) | 54 (56.3) |  |
| Adhesion at 96 h, n (%) | 78 (79.6) | 94 (89.5) | 0.0493 |  | 86 (84.3) | 83 (86.5) | 0.6697 |
| Outgrowth area at 96 h (10⁴×㎛²) | 21.7 ± 1.9 | 29.7 ± 2.1 | 0.0046 |  | 21.0 ± 1.8 | 20.3 ± 1.8 | 0.8124 |
| Outgrowth degeneration, n (%) | 23 (24.5) | 15 (15.9) | 0.0903 |  | 39 (39.0) | 26 (28.0) | 0.1048 |

**Supplemental Table 5. Embryo characteristics and outgrowth outcomes, stratified by blastocyst morphology**

| Blastocyst morphology | Good morphology* | | |  | Poor morphology** | | |
| --- | --- | --- | --- | --- | --- | --- | --- |
|  | Control | Fatty acid | *P* value |  | Control | Fatty acid | *P* value |
| No. of blastocysts, n | 91 | 96 |  |  | 109 | 105 |  |
| Maternal age (y) | 36.1 ± 0.4 | 36.3 ± 0.4 | 0.5569 |  | 36.1 ± 0.4 | 36.1 ± 0.5 | 0.5659 |
| Culture time (h) | 122.8 ± 1.0 | 124.3 ± 1.0 | 0.3192 |  | 133.8 ± 1.0 | 131.8 ± 1.0 | 0.1621 |
| Day 5, n (%) | 68 (74.3) | 64 (66.7) | 0.2267 |  | 30 (27.5) | 41 (39.1) | 0.0735 |
| Day 6, n (%) | 23 (25.3) | 32 (33.3) | 0.2267 |  | 79 (72.5) | 64 (61.0) | 0.0735 |
| Adhesion at 96 h, n (%) | 77 (84.6) | 88 (91.7) | 0.1347 |  | 87 (79.8) | 89 (84.8) | 0.3440 |
| Outgrowth area at 96 h (10⁴×㎛²) | 24.0 ± 2.0 | 32.2 ± 1.9 | 0.0029 |  | 19.2 ± 1.7 | 18.9 ± 1.9 | 0.9186 |
| Outgrowth degeneration, n (%) | 24 (27.0) | 14 (14.6) | 0.0373 |  | 38 (36.2) | 27 (27.6) | 0.1874 |

* Gardner’s criteria; AA, AB, BA, and BB.

** Gardner’s criteria; AC, BC, CA, CB, and CC.

**Supplemental Table 6. Multivariate logistic regression analyses of ongoing pregnancy after single vitrified-warmed blastocyst transfer**

|  | Adjusted odds ratio | 95% CI | *P* value | AUC |
| --- | --- | --- | --- | --- |
| Maternal age* | 0.870 | 0.847–0.894 | < 0.0001 | 0.745 |
| Paternal age* | 1.000 | 0.981–1.019 | 0.9985 |  |
| Previous OR cycles* | 1.049 | 0.995–1.106 | 0.0710 |  |
| Previous ET cycles* | 0.960 | 0.769–1.192 | 0.7166 |  |
| Previous implantation failure* | 0.886 | 0.705–1.118 | 0.3054 |  |
| Endometrial thickness on the day of SVBT (mm) * | 1.041 | 0.994–1.090 | 0.0854 |  |
| Culture time to the expanded blastocyst stage* | 0.966 | 0.956–0.975 | < 0.0001 |  |
| Gardner’s criteria |  |  |  |  |
| ICM Grade A** | Reference | – | – |  |
| Grade B** | 1.087 | 0.865–1.366 | 0.4721 |  |
| Grade C** | 0.771 | 0.550–1.081 | 0.1317 |  |
| TE Grade A** | Reference | – | – |  |
| Grade B** | 0.702 | 0.561–0.879 | 0.0021 |  |
| Grade C** | 0.431 | 0.323–0.576 | < 0.0001 |  |
| Warming solution |  |  |  |  |
| Control** | Reference | – | – |  |
| Fatty acid** | 1.252 | 1.053–1.488 | 0.0109 |  |

OR, oocyte retrieval; ET, embryo transfer; SVBT, single vitrified-warmed blastocyst transfers; ICM, inner cell mass; TE, trophectoderm; CI, confidence interval; AUC, area under the curve. *, continuous variable; **, categorical variable.

Training: Number of rows, 1590; -LogLikelihood, 907.1984; number of parameters, 12; AICc, 1840.63; BIC, 1910.23; AUC, 0.745.

Validation: Number of rows, 1058; -LogLikelihood, 597.7129; number of parameters, 12; AICc, 1221.77; BIC, 1285.96; AUC, 0.752.

Goodness of fit test: χ2 = 484.3137, df = 12, *P* value < 0.0001.

**Supplemental Table 7. Pregnancy outcomes after single vitrified-warmed blastocyst transfers, stratified by maternal age**

|  | Maternal age: < 35 years | | |  | Maternal age: 35–37 years | | |  | Maternal age: 38–40 years | | |  | Maternal age: > 40 years | | |
| --- | --- | --- | --- | --- | --- | --- | --- | --- | --- | --- | --- | --- | --- | --- | --- |
|  | Control | Fatty acid | *P* value |  | Control | Fatty acid | *P* value |  | Control | Fatty acid | *P* value |  | Control | Fatty acid | *P* value |
| Embryo transfer cycles, *n* | 301 | 328 |  |  | 286 | 281 |  |  | 356 | 349 |  |  | 381 | 366 |  |
| Maternal age (years) | 31.7 ± 0.1 | 31.9 ± 0.1 | 0.2562 |  | 36.1 ± 0.0 | 36.0 ± 0.0 | 0.0740 |  | 39.1 ± 0.0 | 39.1 ± 0.0 | 0.2340 |  | 42.3 ± 0.1 | 42.5 ± 0.1 | 0.1028 |
| Paternal age (years) | 34.9 ± 0.3 | 35.1 ± 0.2 | 0.5934 |  | 38.8 ± 0.2 | 38.4 ± 0.3 | 0.3194 |  | 40.8 ± 0.2 | 40.6 ± 0.2 | 0.5157 |  | 43.6 ± 0.3 | 43.9 ± 0.3 | 0.4494 |
| Culture time to the expanded blastocyst stage (h) | 124.1 ± 0.6 | 124.2 ± 0.6 | 0.9241 |  | 124.6 ± 0.7 | 123.5 ± 0.7 | 0.2856 |  | 125.7 ± 0.6 | 125.8 ±0.6 | 0.9217 |  | 128.0 ± 0.6 | 128.4 ± 0.6 | 0.7165 |
| Morphological grade |  |  |  |  |  |  |  |  |  |  |  |  |  |  |  |
| ICM Grade A, *n* (%) | 138 (45.2) | 156 (47.6) | 0.8201 |  | 129 (45.1) | 119 (42.4) | 0.7862 |  | 164 (46.1) | 144 (41.3) | 0.3852 |  | 143 (37.5) | 144 (39.3) | 0.5501 |
| Grade B, *n* (%) | 112 (37.2) | 115 (35.1) |  |  | 100 (35.0) | 105 (37.4) |  |  | 118 (33.2) | 131 (37.5) |  |  | 134 (35.2) | 115 (31.4) |  |
| Grade C, *n* (%) | 53 (17.6) | 57 (17.4) |  |  | 57 (19.9) | 57 (20.3) |  |  | 74 (20.8) | 74 (21.2) |  |  | 104 (27.3) | 107 (29.2) |  |
| TE Grade A, *n* (%) | 114 (37.9) | 131 (39.9) | 0.3159 |  | 106 (37.1) | 108 (38.4) | 0.5991 |  | 118 (33.2) | 111 (31.8) | 0.9303 |  | 98 (25.7) | 94 (25.7) | 0.9385 |
| Grade B, *n* (%) | 99 (32.9) | 90 (27.4) |  |  | 84 (29.4) | 72 (25.6) |  |  | 102 (28.7) | 102 (29.2) |  |  | 102 (26.8) | 102 (27.9) |  |
| Grade C, *n* (%) | 88 (29.2) | 107 (32.6) |  |  | 96 (33.6) | 101 (35.9) |  |  | 136 (38.2) | 136 (39.0) |  |  | 181 (47.5) | 170 (46.5) |  |
| Implantation, *n* (%) | 184 (61.1) | 211 (64.3) | 0.4069 |  | 158 (55.2) | 168 (59.8) | 0.2741 |  | 154 (43.3) | 173 (49.6) | 0.0929 |  | 115 (30.2) | 116 (31.7) | 0.6553 |
| Clinical pregnancies, *n* (%) | 171 (56.8) | 207 (63.1) | 0.1071 |  | 148 (51.8) | 159 (56.6) | 0.2480 |  | 134 (37.6) | 157 (45.0) | 0.0476 |  | 99 (26.0) | 96 (26.2) | 0.9392 |
| Ongoing pregnancies, *n* (%) | 153 (50.8) | 182 (55.5) | 0.2422 |  | 132 (46.2) | 149 (53.0) | 0.1018 |  | 109 (30.6) | 129 (37.0) | 0.0749 |  | 71 (18.6) | 70 (19.1) | 0.8640 |
| Early pregnancy loss, *n* (%) | 13 (7.1) | 4 (1.9) | 0.0123 |  | 10 (6.3) | 9 (5.4) | 0.7282 |  | 20 (13.0) | 16 (9.3) | 0.2810 |  | 16 (13.9) | 20 (17.2) | 0.4856 |
| Miscarriages at the first trimester, *n* (%) | 18 (10.5) | 25 (12.5) | 0.6126 |  | 16 (10.8) | 11 (7.0) | 0.2355 |  | 25 (18.7) | 28 (17.8) | 0.8562 |  | 28 (28.3) | 26 (27.1) | 0.8516 |

The data are shown as mean and standard error of the mean, unless otherwise indicated.

ICM, inner cell mass; TE, trophectoderm.

**Supplemental Table 8. Pregnancy outcomes after single vitrified-warmed blastocyst transfers, stratified by culture time to the expanded blastocyst stage**

|  | Day 4/5 | | |  | Day 6/7 | | |
| --- | --- | --- | --- | --- | --- | --- | --- |
|  | Control | Fatty acid | *P* value |  | Control | Fatty acid | *P* value |
| Embryo transfer cycles, *n* | 872 | 889 |  |  | 452 | 435 |  |
| Maternal age (years) | 37.4 ± 0.1 | 37.2 ± 0.1 | 0.2733 |  | 38.2 ± 0.2 | 38.4 ± 0.2 | 0.4953 |
| Paternal age (years) | 39.7 ± 0.2 | 39.2 ± 0.2 | 0.0746 |  | 40.1 ± 0.3 | 40.6 ± 0.3 | 0.1526 |
| Culture time to the expanded blastocyst stage (h) | 117.7 ± 0.1 | 117.8 ± 0.1 | 0.6274 |  | 141.3 ± 0.2 | 141.6 ± 0.3 | 0.4582 |
| Morphological grade |  |  |  |  |  |  |  |
| ICM Grade A, *n* (%) | 508 (58.3) | 507 (57.0) | 0.8703 |  | 64 (14.2) | 56 (12.9) | 0.7557 |
| Grade B, *n* (%) | 282 (32.3) | 295 (33.2) |  |  | 182 (40.3) | 171 (39.3) |  |
| Grade C, *n* (%) | 82 (9.4) | 87 (9.8) |  |  | 206 (45.6) | 208 (47.8) |  |
| TE Grade A, *n* (%) | 396 (45.4) | 413 (46.5) | 0.4176 |  | 40 (8.9) | 31 (7.1) | 0.6396 |
| Grade B, *n* (%) | 284 (32.6) | 265 (29.8) |  |  | 103 (22.8) | 101 (23.2) |  |
| Grade C, *n* (%) | 192 (22.0) | 211 (23.7) |  |  | 309 (68.4) | 303 (69.7) |  |
| Implantation, *n* (%) | 501 (57.5) | 537 (60.4) | 0.2082 |  | 110 (24.3) | 131 (30.1) | 0.0531 |
| Clinical pregnancies, *n* (%) | 454 (52.1) | 501 (56.4) | 0.0707 |  | 98 (21.7) | 118 (27.1) | 0.0589 |
| Ongoing pregnancies, *n* (%) | 382 (43.8) | 434 (48.8) | 0.0350 |  | 83 (18.4) | 96 (22.1) | 0.1692 |
| Early pregnancy loss, *n* (%) | 47 (9.4) | 36 (6.8) | 0.1202 |  | 12 (10.9) | 13 (9.9) | 0.8027 |
| Miscarriages at the first trimester, *n* (%) | 72 (15.9) | 68 (13.7) | 0.3375 |  | 15 (15.3) | 22 (18.6) | 0.5168 |

The data are shown as mean and standard error of the mean, unless otherwise indicated.

ICM, inner cell mass; TE, trophectoderm.

**Supplemental Table 9. Pregnancy outcomes after single vitrified-warmed blastocyst transfers, stratified by blastocyst morphology**

|  | Good morphology* | | |  | Poor morphology** | | |
| --- | --- | --- | --- | --- | --- | --- | --- |
|  | Control | Fatty acid | *P* value |  | Control | Fatty acid | *P* value |
| Embryo transfer cycles, *n* | 781 | 775 |  |  | 543 | 549 |  |
| Maternal age (years) | 37.3 ± 0.1 | 37.2 ± 0.1 | 0.6435 |  | 38.2 ± 0.2 | 38.1 ± 0.2 | 0.7296 |
| Paternal age (years) | 39.8 ± 0.2 | 39.3 ± 0.2 | 0.0711 |  | 39.9 ± 0.2 | 40.3 ± 0.3 | 0.2939 |
| Culture time to the expanded blastocyst stage (h) | 120.6 ± 0.3 | 120.4 ± 0.3 | 0.8020 |  | 133.3 ± 0.5 | 132.9 ± 0.5 | 0.6448 |
| Morphological grade |  |  |  |  |  |  |  |
| ICM Grade A, *n* (%) | 545 (69.8) | 527 (68.0) | 0.4476 |  | 27 (5.0) | 36 (6.6) | 0.4582 |
| Grade B, *n* (%) | 236 (30.2) | 248 (32.0) |  |  | 228 (42.0) | 218 (39.7) |  |
| Grade C, *n* (%) | – | – |  |  | 288 (53.0) | 295 (53.7) |  |
| TE Grade A, *n* (%) | 434 (55.6) | 443 (57.2) | 0.5268 |  | 2 (0.4) | 1 (0.2) | 0.6208 |
| Grade B, *n* (%) | 347 (44.4) | 332 (42.8) |  |  | 40 (7.4) | 34 (6.2) |  |
| Grade C, *n* (%) | – | – |  |  | 501 (92.3) | 514 (93.6) |  |
| Implantation, *n* (%) | 453 (58.0) | 480 (61.9) | 0.1134 |  | 158 (29.1) | 188 (34.2) | 0.0676 |
| Clinical pregnancies, *n* (%) | 407 (52.1) | 453 (58.5) | 0.0119 |  | 145 (26.7) | 166 (30.2) | 0.1958 |
| Ongoing pregnancies, *n* (%) | 347 (44.4) | 399 (51.5) | 0.0054 |  | 118 (21.7) | 131 (23.9) | 0.4015 |
| Early pregnancy loss, *n* (%) | 46 (10.2) | 27 (5.7) | 0.0112 |  | 13 (8.2) | 22 (11.7) | 0.2857 |
| Miscarriages at the first trimester, *n* (%) | 60 (14.7) | 55 (12.2) | 0.2798 |  | 27 (18.6) | 35 (21.1) | 0.5875 |

The data are shown as mean and standard error of the mean, unless otherwise indicated.

ICM, inner cell mass; TE, trophectoderm

*Gardner’s criteria; AA, AB, BA, and BB.

** Gardner’s criteria; AC, BC, CA, CB, and CC.

**Supplemental Table 10. Association between blastocyst outgrowth and embryo characteristics**

|  | *P* value |
| --- | --- |
| Maternal age* | 0.0080 |
| Culture time to the expanded blastocyst stage* | 0.0046 |
| Inner cell mass** | 0.0022 |
| Trophectoderm** | < 0.0001 |
| Warming solutions** | 0.0331 |

*, continuous variable; **, categorical variable.

**Supplemental Table 11. Association between ongoing pregnancy rate and embryo characteristics**

|  | *P* value |
| --- | --- |
| Maternal age* | <0.0001 |
| Paternal age* | <0.0001 |
| Previous oocyte retrieval cycles* | 0.0019 |
| Previous embryo transfer cycles* | 0.0030 |
| Previous implantation failure* | 0.0098 |
| Endometrial thickness on the day of SVBT (mm) * | 0.0002 |
| Body mass index* | 0.7606 |
| Culture time to the expanded blastocyst stage* | <0.0001 |
| Inner cell mass** | <0.0001 |
| Trophectoderm** | <0.0001 |
| Warming solutions** | 0.0091 |

*, continuous variable; **, categorical variable.
